# Guppies Show Behavioural but Not Cognitive Sex Differences in a Novel Object Recognition Test

**DOI:** 10.1371/journal.pone.0156589

**Published:** 2016-06-15

**Authors:** Tyrone Lucon-Xiccato, Marco Dadda

**Affiliations:** Dipartimento di Psicologia Generale, Università di Padova, Padova, Italy; University of Vienna, AUSTRIA

## Abstract

The novel object recognition (NOR) test is a widely-used paradigm to study learning and memory in rodents. NOR performance is typically measured as the preference to interact with a novel object over a familiar object based on spontaneous exploratory behaviour. In rats and mice, females usually have greater NOR ability than males. The NOR test is now available for a large number of species, including fish, but sex differences have not been properly tested outside of rodents. We compared male and female guppies (*Poecilia reticulata*) in a NOR test to study whether sex differences exist also for fish. We focused on sex differences in both performance and behaviour of guppies during the test. In our experiment, adult guppies expressed a preference for the novel object as most rodents and other species do. When we looked at sex differences, we found the two sexes showed a similar preference for the novel object over the familiar object, suggesting that male and female guppies have similar NOR performances. Analysis of behaviour revealed that males were more inclined to swim in the proximity of the two objects than females. Further, males explored the novel object at the beginning of the experiment while females did so afterwards. These two behavioural differences are possibly due to sex differences in exploration. Even though NOR performance is not different between male and female guppies, the behavioural sex differences we found could affect the results of the experiments and should be carefully considered when assessing fish memory with the NOR test.

## Introduction

Behavioural paradigms that exploit spontaneous object preference are widely used to study memory and its impairments in animals [1]. The novel object recognition (NOR) test, also called the novel object preference test, is a non-reinforcement based paradigm that relies on spontaneous exploratory behaviour [2]. In its classical form, developed for rats, the NOR test consists of two phases [2, 3]. Subjects initially undergo a one-trial familiarisation with one object or two identical objects in an open field; after an established time interval, subjects attend a test phase that exposes them simultaneously to the familiar object and to a novel one. During the test phase, subjects are expected to interact mainly with the novel object because of exploratory behaviour. By measuring the preference for the novel object over the familiar one, it is possible to assess NOR performance [2]. The NOR test has emerged as one of the most popular paradigms to assess memory in rats and other rodents, such as mice and hamsters [3, 4, 5, 6], because it is quick to administer and relatively simple. Moreover, the NOR test represents a promising paradigm for comparative research [1] since it can be successfully adapted to very different mammalian and avian species, such as monkeys [7, 8], dogs [9], cats [10], horses [11], ravens [12], and parrots [13].

In rats and mice, females usually have greater NOR performance than males ([14, 15, 16, 17] but see [18]). According to some authors, this cognitive sex difference is due, at least in part, to the greater attention to objects and their features expressed by female rodents [16, 17]. For example, Bettis and Jacobs [16] found female mice outperformed males in NOR test if the two objects were very similar, but not if the two objects differed to a great extent. Such hypothesis is supported also by the fact that female mammals show greater attention to objects and greater ability in encoding object features in many different tests. For example, female humans, mice, rats and kangaroo rats (*Dipodomys merriami* and *D*. *microps*) exploit objects as local landmarks to orientate during spatial navigation more than males do [19, 20, 21, 22]. In humans and rats, females are more sensitive to position changes in an array of familiar objects [23, 24]. In dogs, females note changes in the size of a familiar object, but males apparently do not [25]. Although there are few studies outside of mammalian species, there is evidence that sex differences in object encoding may also subsist in birds. Having to discriminate between two objects that differ both in features (such as colour) and in position, female domestic chicks preferentially attend to features, whereas males encode position [26]. The possibility exists that sex differences in tests that require object encoding are a widespread phenomenon across vertebrates.

In the last decade, fish have become increasingly popular as an animal model for the study of memory, and the NOR test has been shown to be available for juvenile guppies [27] and zebrafish [28, 29, 30]. However, it is unknown whether sex differences in NOR exist in fish. Curiously, researchers often do not consider or even report the sex of the subjects while studying NOR in fish [27, 28, 29, 30, 31]. If sex differences in object recognition are also present in fish, this may confound the interpretations of past and future studies. Here, we examined the existence of sex differences in NOR test in a fish, the guppy (*Poecilia reticulata*). The guppy is a fish species commonly used for cognitive experiments [32], and perhaps the most studied in the field of cognitive and behavioural sex differences [33, 34, 35, 36, 37].

We tackled the problem of sex differences from two different angles. The first goal of our work was to study cognitive sex differences in NOR performance. We expected sex differences in NOR performance because a previous study found greater female performance in a test that required object discrimination [34]. Moreover, a peculiar aspect of guppy biology suggests the existence of sex differences in NOR performance. During mate choice, female guppies use visual characteristics, such as body size, tail length, and body colouration, to carefully evaluate male attractiveness [38]. In particular, a large number of studies have revealed an amazing ability of female guppies to discriminate among available males on the basis of subtle differences in size, shape, number, hue and intensity of their colour spots [38, 39, 40]. Females also learn, memorise, and remember these features in order to provide an experiential base for successive mating decisions [41, 42]. By contrast, male guppies do not perform mate choice [38]. The high cognitive requirements for mate choice suggest that selection should favour the evolution of enhanced ability for discerning, learning, memorising and discriminating stimulus features in female guppies [43, 44]. Accordingly, we expected (*i*) female guppies to show greater performance in NOR test (that is greater preference for the novel over the familiar object).

Sex differences in cognitive tests may also emerge from the different behaviour of males and females [45]. Therefore, the second goal of our study was to investigate behavioural sex differences in the NOR test. In the case of the NOR test, sex difference in exploratory behaviour is a possible source of variance between the sexes. In guppies, males are notably bolder [46, 47] and more active than females [47]. Male guppies are therefore expected to be more prone to approach and explore objects as previously reported for closely related poeciliid species [48]. Accordingly, in our NOR test we expected (*ii*) male guppies to exhibit more general attraction toward the objects and (*iii*) to approach the novel object earlier than females.

Since a previous NOR experiment on guppies only used juveniles [27], we also investigated whether or not the NOR test would be suitable to examine object recognition memory in adult guppies.

## Materials and Methods

### Ethical statement

The experiment complied with the law of the country (Italy) in which it was performed (Decreto legislativo 4 Marzo 2014, n. 26). The experimental procedures have been approved by Università di Padova Ethical Committee (protocol n. 33/2015). In our experiment, we exposed fish to harmless objects and observed their spontaneous exploratory behaviour. None of the fish were subjected to manipulations, anaesthesia, euthanasia, or any form of deprivation. Fish were maintained in tanks enriched with natural plants and social companions and were fed ab libitum. After the experiment, subjects were released in maintenance tanks and kept only for breeding purposes.

### Experimental subjects

The experimental subjects were 16 male and 16 female guppies (5–6 months old) reared in our laboratory. These guppies descended from wild guppies caught in 2002 in the lower course of the Tacarigua River, in Trinidad and Tobago. Since collection guppies were maintained in our laboratory in 100 × 70 × 54 cm plastic tanks in large mixed-sex groups with a sex ratio of 1:1. In the tanks, we added gravel to a height of 3 cm, 10 *Hygrophila corymbosa* plants, and approximately 24 dm^3^ of Java moss (*Taxiphyllum barbieri*) as refuge. The water was maintained at 26 ± 1°C and was continuously filtered and aerated to keep conditions constant. Fluorescent lamps supplied illumination from 7:30 to 19:30 each day. The fish were fed commercial food flakes (Fioccomix, Super Hi Group, Ovada, Italy) and live *Artemia salina* nauplii, twice in the morning and once in the afternoon. Experimental subjects were randomly selected from the maintenance tanks. Each subject was tested only once.

### Stimuli and experimental tanks

**Fig 1:**
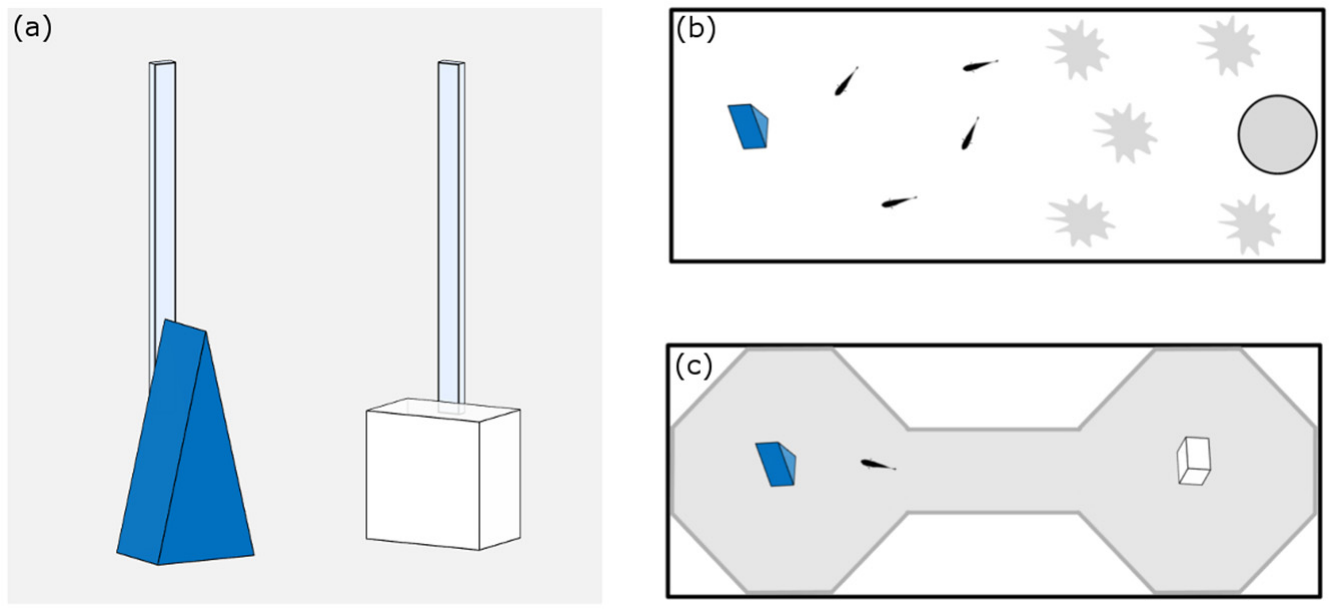
Schematic representation of stimuli and apparatuses. (a) Blue pyramid and the white parallelepiped used as stimuli; (b) familiarisation tank, half occupied by plants and water filter; (c) two-chambers tank used to asses NOR performance in the test phase.

We used two objects as stimuli in the experiment, a white plastic parallelepiped (4 × 4 × 2 cm) and a blue plastic pyramid (base: 4 × 3 cm, height: 7 cm; Fig 1a). We used four identical copies of each object. A transparent plastic rod fixed to each object allowed its insertion into the experimental tanks, 10 cm under the water’s surface.

The familiarisation phase was performed in 50 × 20 × 32 cm tanks (Fig 1b) filled with 25 cm of water. The bottom of each tank was made of natural gravel. One half of the tank housed a water filter and natural vegetation (approximately 4 dm^3^ of Java moss). The other half was used to present the object to be familiarised (hereafter ‘familiar’) during familiarisation. The longer walls of the tank were covered with green plastic, and fluorescent lamps positioned above the middle of the tank provided illumination.

The test phase was performed in a two-chamber apparatus built with grey plastic material inside a 50 × 20 × 32 cm tank (Fig 1c). Above the central corridor (14 × 6 cm) that connected the two chambers, two fluorescent lamps, oriented toward the chambers, provided illumination. Pilot experiments suggested that guppies used the central corridor as a safe place, especially at the beginning of the test. This is in line with previous works that revealed guppies, and other fish species, avoid the middle area of the open field when inserted in experimental arenas [49, 50, 51]. We used four identical test tanks to simultaneously observe four subjects. Before the test, the two objects were inserted in the two chambers: the familiar object and the complementary (hereafter ‘novel’) object, with a balanced position across subjects. A digital video camera (Canon Legria FS200, 25 fps) mounted one meter above the tank recorded the experiments.

### Testing procedure

As the guppy is a social species, we exposed the subjects to the familiarisation phase in groups of four (two males and two females) to avoid social deprivation. Overall, we observed eight groups of subjects. Six days before familiarisation, we moved each group of four subjects from the maintenance tanks to a familiarisation tank to habituate. On the seventh day, between 11:00 and 14:00, we gently inserted the familiar object into the empty half of the tank for familiarisation. To control for innate preference, half of the groups familiarised with the parallelepiped and half with the pyramid. Groups were randomly assigned to one of two familiarisation time lengths (one hour or three hours). At the end of the familiarisation, the object was removed from the tank and the subjects were left undisturbed.

The test phase took place after an interval of one day for half the groups, and six days for the remaining groups. The duration of the familiarisation and the interval were balanced between subjects. Each subject was netted from the familiarisation tank and moved into a small plastic jar filled with water. The jar was then gently inserted in the central corridor of the test tank to let the subject swim out of the jar. Subject behaviour was recorded for 12 minutes. We used two different familiarisation time lengths and two different interval lengths to examine the suitability of NOR test in adult guppies.

During the habituation and interval, subjects were fed three times a day, as they had been in the maintenance tanks. On the familiarisation day, subjects were fed in excess twice, once in the morning and once in the evening.

### Data collection and analysis

We observed subjects’ behaviour from the video recordings. Using computer software (Ciclic Timer), we measured the proportion of time subjects spent in each of two chambers and in the central corridor during each two minute interval in the experiment. The proportion of time spent in the central corridor was used to study sex differences in exploratory behaviour (prediction *ii*). Subjects with greater exploratory behaviour were expected to spend less time in the central corridor. In each block of two minutes, the preference for the novel object was computed as time spent in the chamber with the novel object divided by the sum of the time spent in the chamber with the familiar object and the time spent in the chamber with the novel object. This variable was considered as measure of NOR performance in the comparison between the sexes (prediction *i*). The use of repeated measures analysis across the blocks of time allowed to study the temporal pattern of the preference for the novel object (prediction *iii*).

Statistical analysis was performed in R (R Core Team, version 3.0.2). Data available in S1 File. All statistical tests were two-tailed, and the significance threshold set was at *P* = 0.05. As the variables were proportions, we always performed an arcsine square root transformation to meet normality assumptions [52]. In the text, data are expressed as *M* ± *SD* percentages. To test whether the preference for the novel object was greater than chance, we used a one-sample *t* test against the mean of a random choice (0.5). The preference for the novel object and the proportion of time that subjects spent in the central corridor were then studied with linear mixed-effects models (LMMs; using the ‘lme’ function) fitted with block of minutes, sex, familiarisation and interval length as fixed effects and with the subject’s ID as random effect to account for repeated measures. As all interactions of second and third order were not significant (*P* > 0.1), we fitted a reduced model with first-order interaction only. To compare males’ and females’ preferences for the novel object in the periods when both expressed a significant preference, we used an independent-samples *t* test. In case of no significant differences between males and females, the performance of the two sexes is likely to be equal. The null hypothesis significance testing permits to identify differences between groups, but is mathematically unable to test if two groups are equal [53, 54]. This raises several issues of the interpretation of null results such as limited statistical power. Thus, if our analysis did not reveal sex difference, we performed a Bayesian analysis that allowed us to evaluate the hypothesis that the performance of males and females was equal, even in the case of small sample size [53, 54]. We computed an approximated Bayes factor (*BF*) using the Bayesian information criterion (BIC) of a linear model fitted with (BIC_sex_) and without (BIC_null_) the effect of sex: *BF* = exp((BIC_sex_—BIC_null_) / 2 [55, 56]. As an example, if *BF* = 5, our data were five times more likely to fit the model without the effect of sex than the model with the effect of sex. One female (in the group with 1-hour familiarisation and a 1-day interval) died due to unknown causes during the interval between familiarisation and the test phase.

## Results

**Fig 2:**
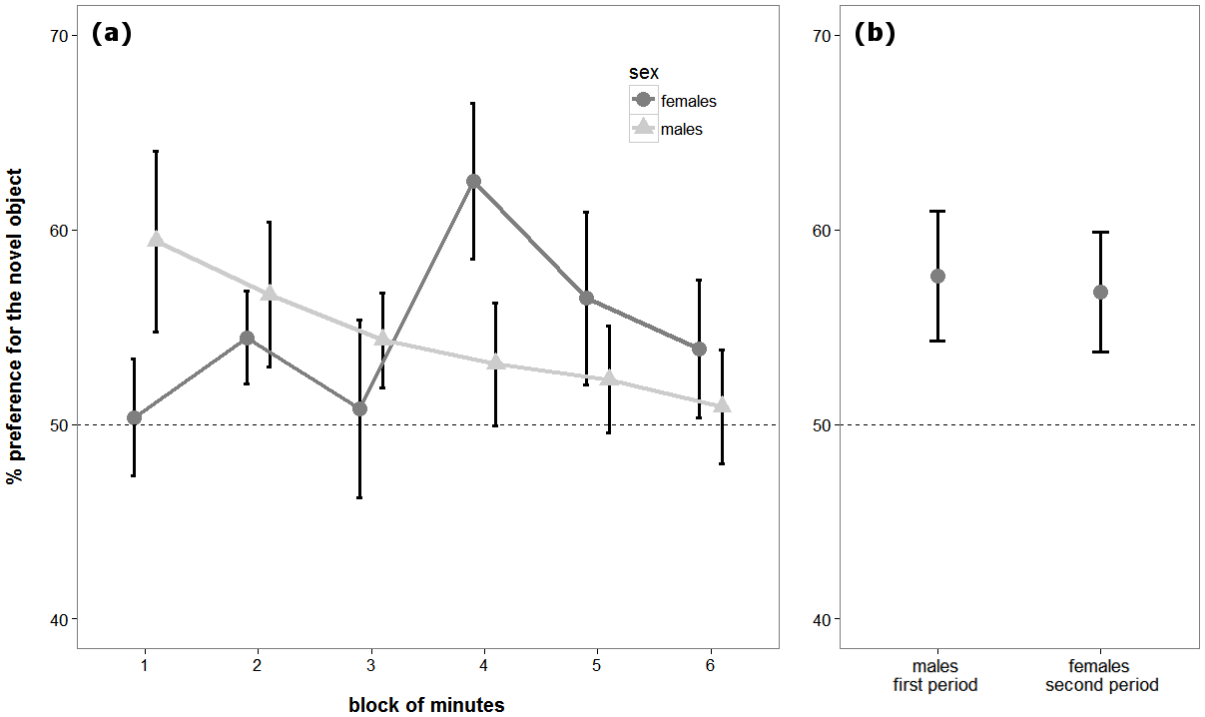
Comparison of NOR performance of male and female guppies. Preference for the novel object (a) during the full length of the experiment (12 minutes subdivided in six blocks) and (b) in the two periods of the experiment in which the two sexes expressed significant preference for the novel object (males: minutes 1 to 6; females: minutes 7 to 12). Data points represent *M* ± *SEM* percentage preference for the novel object.

Overall, both male and female subjects demonstrated a significant preference for the novel object (54.59 ± 9.11%, one-sample *t* test: *t*_30_ = 2.771, *P* = 0.010). In the LMM on the preference for the novel object, we found no significant effect for the block of minutes (*F*_5,135_ = 0.900, *P* = 0.483), familiarisation length (*F*_1,24_ = 3.761, *P* = 0.064), or interval length (*F*_1,24_ = 0.185, *P* = 0.671). Sex did not significantly influence the preference for the novel object (*F*_1,24_ = 0.051, *P* = 0.824, *BF* = 5.513). However, there was a significant interaction between sex and block of minutes (*F*_5,135_ = 2.664, *P* = 0.025; Fig 2a). Sex by block of minutes interaction was significant also in a reduced model fitted with these two effects only (*F*_5,145_ = 2.678, *P* = 0.024). Graphical inspection of Fig 2a suggested that males expressed preference for the novel object at the beginning of the experiment, which subsequently decreased with time; females expressed preference for the novel object only in the second half of the experiment. No other significant interactions were found in the model.

We performed further analysis for the first and second halves of the experiment separately (minutes 1 to 6 and 7 to 12, respectively) to study the significant interaction in the previous LMM (sex by block of minutes). In the first period of the experiment, males significantly preferred the novel object (56.79 ± 11.90%, one-sample *t* test: t_15_ = 2.220, *P* = 0.042), but females did not (51.87 ± 9.45%, *t*_14_ = 0.785, *P* = 0.446; Fig 2b). In the second period of the experiment, we observed the opposite situation: females significantly preferred the novel object (57.62 ± 12.88%, *t*_14_ = 2.240, *P* = 0.042), but males did not (52.10 ± 11.90%, *t*_15_ = 1.035, *P* = 0.317; Fig 2b). A direct comparison between the males’ preference in the first period and females’ preference in the second period revealed no difference between the two sexes in terms of NOR performance (independent-samples *t* test: *t*_29_ = 0.190, *P* = 0.851, *BF* = 5.462; Fig 2b; Table 1).

**Table 1:** Descriptive statistics (*M* ± *SD*) of the preference for the novel objects of males (minutes 1–6) and females (minutes 7–12).

	1-day interval	6-day interval
**1-hour familiarisation**	males: 61.24 ± 18.95%	males: 54.57 ± 10.02%
	females: 67.52 ± 17.13%	females: 58.00 ± 17.74%
**3-hour familiarisation**	males: 52.92 ± 5.42%	males: 58.55 ± 12.72%
	females: 54.88 ± 8.89%	females: 53.27 ± 8.37%

**Fig 3:**
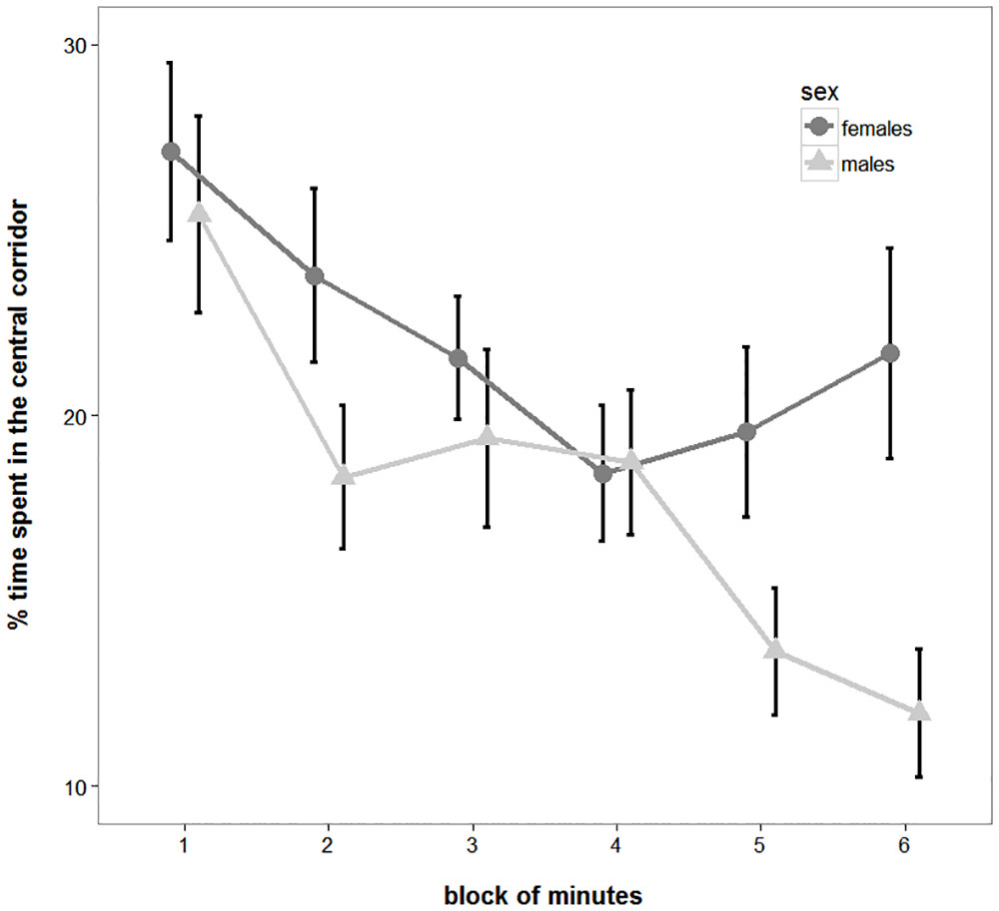
Comparison of object attraction of male and female guppies. Percentage of time (*M* ± *SEM*) male and female guppies spent in the central corridor during the test phase of the experiment subdivided in the six two-minute blocks. The smaller the percentage of time spent in the central corridor, the higher the attraction for the objects.

To study exploratory behaviour, we analysed time spent in the central corridor. On average, subjects spent 19.90 ± 6.15% of their time in the central corridor. The LMM on this variable revealed that proportion of time spent in the central corridor significantly decreased as the experiment went on (*F*_5,135_ = 6.643, *P* < 0.001; Fig 3). In the model, there was also a significant effect of sex: females spent more time in the central corridor than males did (females: 22.02 ± 6.61%; males: 17.92 ± 6.12%; *F*_1,24_ = 4.564, *P* = 0.043; Fig 3). Moreover, subjects that were tested after a 6-day interval spent significantly more time in the central corridor than did those tested after a 1-day interval (1 day: 16.83 ± 5.48%; 6 days: 22.78 ± 5.41%; *F*_1,24_ = 9.525, *P* = 0.005). There was no significant effect of familiarisation length (*F*_1,24_ = 0.419, *P* = 0.524), nor any significant interaction.

## Discussion

### Suitability of the NOR test for adult guppies

We compared male and female guppies in a NOR test to investigate the existence of sex differences. To date, this is the first investigation of sex differences in the NOR test in fish. This is also the first work that studied NOR in adult guppies. Subjects proved able to discriminate the familiar object from the novel object by expressing a preference for the latter. Therefore, adult guppies are able to spontaneously learn and memorise the characteristics of a novel object after a single exposure, allowing proper use of the NOR test. The neophilic response toward the novel object of adult guppies is similar to those exhibited by other species in the NOR test, including rats [2], mice [16], and zebrafish [28]. However, the only study that observed NOR in guppies used juveniles and reported very different behaviour. Miletto Petrazzini and colleagues [27] found that juvenile guppies perform NOR but avoid the novel object and prefer the familiar one during test phase as previously observed by May and colleagues in adult zebrafish [29]. This difference could be due to methodological differences between the two studies as well as to behavioural differences between juvenile and adult guppies. In the study of Miletto Petrazzini and colleagues [27], objects were conspicuously coloured and much larger than the subjects. This could have repulsed rather that attracted the subjects. Alternatively, juvenile guppies can be shier than adults in presence of novel objects [28].

In our experiment, guppies showed no NOR performance reduction in the 6-day interval relative to the 1-day interval. In contrast, a previous study in fish has revealed NOR performance drops after 96 hours [31]. This incongruence could be explained by Braida and colleagues [31]’s use of bi-dimensional figures as stimuli, while we used solid objects in the present study. Indeed, with training procedures, discrimination learning is improved by using solid stimuli instead of bi-dimensional ones [57]. Our data revealed that the comparison between experiments that adopt different kinds of stimuli should be made with great caution in spontaneous behavioural tests such as the NOR. Interestingly, we also found no difference in NOR performance due to the familiarisation length, but the familiarisation lengths adopted in this study (30 and 60 minutes) were much longer than the ones commonly adopted in rodents [2], and subjects probably had enough time to acquire information on object features in both conditions.

Proportion of time spent in the central corridor was higher at the beginning of the experiment, but decreased quickly. Since guppies tend to avoid open fields in a novel experimental arena [49, 50, 51], the time in the central corridor is likely to reflect avoidance of the two chambers with the objects. Therefore, such decreasing trend indicated that guppies initially approached the two objects carefully, but gradually increased interaction because they habituated to the objects or to the test apparatus. Furthermore, we found an effect of the interval length on proportion of time spent in the central corridor. Guppies tested after a 6-day interval remained more in the central corridor than those tested after a 1-day interval. This effect could be due to the fact that 6 days after the familiarisation phase guppies were somewhat shier in the presence of the two objects. Both the effect of time within the test phase and the effect of interval length are not likely to affect the measure of NOR performance computed as relative preferences for the novel object over the familiar object. However, we recommended considering these two parameters when designing NOR experiments.

### Cognitive sex differences

The overall analysis on the preference for the novel object did not reveal any sex difference in NOR performance. This result was further confirmed by the analysis that accounted for sex-specific exploration timing: male preference for the novel object at the beginning of the experiment was equal to female preference in the second period of the experiment. In both cases, Bayesian analysis allowed us to evaluate the hypothesis that males and females had the same performance irrespective from sample size and found ‘substantial’ support for it [58]. Although a single experiment is not enough to draw clear conclusions about cognitive abilities of a species, our result seems to suggest that male and female guppies have equal ability in memorising objects’ features and use such information thereafter to recognise and discriminate a familiar object from a novel one.

Since we have failed to find, in fish, the sex differences in object encoding often found in mammals, and since other authors have failed the same goal when studying birds [59, 60], this kind of sex differences might not to be a shared characteristic of vertebrates’ systems for object encoding. It is possible that sex differences in object encoding have evolved only in vertebrates with sophisticated nervous systems, or perhaps only in some mammal [14, 15, 16, 17] and bird species [26] as a consequence of specific ecological demands. It should be said that this lack of cognitive sex differences was unexpected for at least two reasons (prediction *i*). The first reason is that in previous studies female guppies have showed more refined discrimination and attention than males [34]. However, the cognitive functions involved in the latter test may be different and independent from that underlying performance in our NOR test. Indeed, performances among different cognitive tests are often unrelated in nonhuman animals [61]. According to some authors, this suggests the existence of several independent cognitive functions whose activation is context- or task-specific [35, 62].

The second reason for expecting a cognitive sex difference in the NOR test, was that female guppies, but not males, are able to operate sophisticated mating decisions based on subtle male body characteristics (see [38, 40]). As a consequence, selection for enhanced learning, mnemonic, and discrimination abilities [43, 44] is expected to be stronger in females. Contrary to our prediction, it is possible that sex-specific selection for these cognitive abilities does not exist. For example, sexual selection might act on the same direction and intensity on male cognition as well. Although nowadays it is difficult to support this hypothesis, a recent study showed that male guppies make adaptive mating decisions by observing rivals’ colouration [63]. Such behaviour raises the possibility that males are also selected for those cognitive functions that allow evaluation of male colouration. Another possibility is that, even if the sex differences in selective pressures we hypothesised do exist, the cognitive functions targeted by selection lack of evolvability or lack the possibility to evolve in a sex-specific manner. In support of this possibility, several lines of evidence have demonstrated that the mechanisms regulating object perception, representation and encoding are extremely conserved in vertebrates [64, 65, 66], suggesting low evolvability of the underlying cognitive functions.

Despite our data suggests that male and female guppies’ NOR performance was similar, there were some factors which we were not able to control in the experiment. Although our experiment had the sensitivity to detect two behavioural sex differences (see next section), it is possible that sex difference in NOR performance exists, but that it is much smaller than behavioural differences in NOR or than cognitive differences in other tasks [34]. Alternatively, it is possible that our result has been confounded by sex differences in the strategy used by males and females to solve the task, such as encoding different cues (shape, size, colour) to recognise the objects. Multiple converging lines of evidence from different experiments are therefore required to confirm the presence of a sex similarity in guppy’s NOR ability.

### Behavioural sex differences

Aside from the lack of sex difference in NOR performance, it is worth noting that the behaviour of male and female guppies was different during the test. The analysis of the proportion of time subjects spent in the central corridor of the test apparatus (where no objects were placed) supported prediction *ii*. Males spent less time than females in the central corridor, as they were more attracted by the two objects or more prone to leave the safe place in the middle of the apparatus [49, 50, 51]. This effect is in line with previous studies, in which male poeciliids were more likely to approach and explore objects [48] and faster to emerge from a safe place into a novel environment [46].

The analysis of the temporal pattern of preference revealed a sex difference also in exploratory timing. As in prediction *iii*, males’ preference for the novel object was higher at the beginning of the experiment but rapidly decreased in the following minutes. Conversely, females expressed a preference for the novel object only in the second period of the experiment, and their performance rapidly decreased in the remaining minutes as well. It has been pointed out that the exploratory behaviour of fish toward novel objects occurs very quickly, and that, subsequently, subjects interact randomly with familiar and novel objects [28]. The temporal pattern observed in our experiment apparently aligns with this previous finding, as guppies expressed a preference for the novel object for very few minutes and then reduced their interactions. However, males and females showed this passing preference in different phases of the experiment. This sex difference could lead to spurious results in novel object experiments with fish if the sex of the subject is not considered. For example, in rodents very often the preference for the novel object is evaluated for a couple of minutes [67]. By using a similarly short test duration in fish, we run the risk of measuring only male NOR performance.

## Conclusions

To summarise, our results align with growing evidence that male and female guppies have similar abilities in solving many cognitive tests [35, 68, 69]. Both males and females showed NOR, but the preference for the novel object was expressed with different timing by the two sexes, probably because of sex differences in exploration. The sex of the fish should be therefore carefully considered to avoid potential erroneous results in NOR experiments.

## Supporting Information

S1 File Data. Preference for the novel object and time in the central corridor of the guppies observed in the experiment.
(XLSX)
